# The Digestive Microbiome Diversity of the Least Killifish, *Heterandria formosa* , and Its Implications for Host Adaptability to Varying Trophic Levels

**DOI:** 10.1111/1758-2229.70164

**Published:** 2025-08-12

**Authors:** Benjamin D. Pluer, Joseph Travis

**Affiliations:** ^1^ Department of Biological Science Florida State University Tallahassee Florida USA

**Keywords:** aquatic microbiology, bioremediation, climate change microorganisms, community assembly, environmental signal/stress responses, functional genomics, microbial ecology, microorganisms in the digestive system

## Abstract

Symbiotic microbes, in associations with aquatic hosts, aid in the acquisition of nutrients, breakdown xenobiotics, and contribute to immune system function. If associations with microbial communities facilitate host adaptation to different ecosystems, understanding the important ecological factors that act as drivers of differences among conspecific populations' microbiomes can help conservation efforts to promote beneficial interactions between fish and their microbiome for freshwater fish species facing rapid environmental changes. Here we describe the microbial communities in the gut of a freshwater fish, *Heterandria formosa* , in spring habitats using 16S rRNA sequencing. We quantified microbiota composition and diversity among springs ranging from oligotrophic to near eutrophic to determine the extent to which the microbiota are associated with different environmental conditions. We found higher microbial richness at sites with lower nutrient load stress. At more eutrophic sites, we detected the potential for increased metabolic capacity for pollutant degradation in the associated microbiota. We noted greater phylogenetic similarity between more environmentally similar sites, supporting previous evidence that the microbiota of freshwater fish is influenced by site water chemistry. Our findings bring to light microbial taxa and pathways that might play critical roles in the bioremediation of stressful environmental conditions.

## Graphical abstract

Investigations of environmental factors influence on the gut microbiome of the least killifish reveal high variability in gut microbiomes between disparate sites and higher microbial diversity in healthier habitats as reported by the Florida Department of Environmental Protection. Findings suggests specific microbial taxa that may aid in bioremediation and highlight how microbial communities may help fish adapt to environmental stress, offering implications for conservation and biodiversity management in freshwater ecosystems.

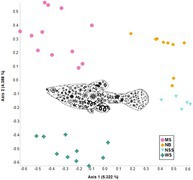

## 1 Introduction

Microbial communities can be found in almost every niche on the planet (Wu et al. 2009; McFall‐Ngai et al. 2013). They drive global biogeochemical cycles and build co‐evolved associations with plant and animal hosts (Cordero et al. 2021; Schloss and Handelsman 2004). Symbiotic autochthonous microbes play critical roles in their associations with aquatic hosts and are often maintained as distinctive communities separate from the microbes present in their habitats and diet (Bledsoe et al. 2016; Yan et al. 2016). These symbiotic microbes aid the host in the acquisition of nutrients, the breakdown of xenobiotics into less harmful compounds, and in modulation of the host's immune system (Kuz'mina 2015; Ramírez and Romero 2017; Sanders et al. 2015). Microbial communities in the digestive tract of younger, healthier fish are particularly important because these fish maintain higher levels of microbial diversity (Baldo et al. 2017; Biagi et al. 2016; Smith et al. 2017).

Aquatic environments are an ideal medium for examining the formation and function of symbiotic microbial associations because the animals living in them are constantly exposed to pathogenic and opportunistic organisms that might not be useful to their hosts (Mohammed and Arias 2015). In particular, freshwater habitats, which cover less than 1% of Earth's surface, play host to nearly 33% of vertebrate species and 10% of all species (Strayer and Dudgeon 2010). These species, including fish, are in constant contact with their freshwater habitat and their digestive tract is an open system constantly exposed to their surrounding environment (Gomez et al. 2013; Romero and Navarrete 2006; Egerton et al. 2018). The microbial communities that establish themselves in the digestive tract of aquatic organisms may come from a variety of sources and establish themselves at different times in the life history of the host (Nayak 2010; Forberg et al. 2016; Bolnick et al. 2014; Ikeda‐ohtsubo et al. 2018). Microbial communities may be acquired through vertical transmission or through more traditional horizontal transmission pathways, primarily through environmental uptake (Shapira 2016; Zhao et al. 2019).

Recent studies attempting to determine the drivers of differences in microbial communities between organisms at the individual and population levels, and the potential benefits of those resident microbiota, have often neglected to examine the ecological consequences of the microbial associations (Greyson‐Gaito et al. 2020; Vatsos 2017). Little is known about the influence of the digestive tract microbiota on an individual's ability to compete for resources or on its capacity to withstand various environmental stressors (Hawkins and Crawford 2018; Hanage 2014; Koskella and Vos 2015).

This gap in our knowledge is particularly striking in the face of declining biodiversity and increasing rates of extinction in North American freshwater fish (Burkhead 2012; WWF 2022). Environmental changes, including climate warming and anthropogenic‐driven nutrient loading, are occurring so rapidly that organisms might struggle to evolve phenotypes suitable for novel conditions at a sufficiently rapid rate (Quintero and Wiens 2013; Neuman et al. 2016; Franks and Hoffmann 2012; Jian Li et al. 2017). However, the recognition that the holobiont, which is a host and its associated microbes, can be a unit of selection in evolution has opened up new perspectives on the capacity of organisms to adapt to rapid environmental change (Zilber‐Rosenberg and Rosenberg 2008; Mouchet et al. 2012; Nayak 2010).

We define local adaptation, in this context, as the ability of a population to adjust biologically to local conditions in order to sustain a viable population with a positive growth rate. This can occur either through genetic differences among host populations or differences in microbiota between individuals from different host populations. Examples of apparent adaptation through changes in the microbiome have been described in digestive tract microbial communities facilitating detoxification of toxic plant secondary compounds (Kohl et al. 2014) niche expansion (Nougué et al. 2015), and pest invasion (Brown et al. 2014).

The role of the microbiome in facilitating local adaptation is enhanced by the ability of microbes to transfer genes horizontally. Horizontal gene transfer means that closely related strains can differ in the presence or absence of thousands of accessory genes and consequently have the potential to adapt more readily to rapid changes in environmental conditions than do their hosts (Prosser et al. 2007). In addition, the phenotypic plasticity of the gut microbiota has the potential to contribute to the adaptability of their host and play a pivotal role in aiding aquatic hosts to acclimate to rapidly changing environmental variation (Alberdi et al. 2016). If associations with adapted microbial communities facilitate host and ecosystem adaptation under rapidly changing environmental conditions, a greater understanding of how to promote the beneficial interactions between freshwater fish and their microbiome has the potential to aid in comprehensive conservation efforts (Hutchins and Fu 2017).

The value of the microbiome of aquatic organisms in the abatement of stress associated with eutrophic conditions remains understudied (Bledsoe et al. 2016). This is an important question for freshwater fishes, which face an increasing challenge from anthropogenic nutrient enrichment of rivers and lakes and increased levels of chemical pollution (Villéger et al. 2017; Sehnal et al. 2021; Horka et al. 2023). In this light, it is surprising that so little is known about the microbiomes of fishes. Of the over 33,000 fish species, however, studies have focused on model and aquacultural fish species and on economically valuable aquatic animals (Colston and Jackson 2016; Yukgehnaish et al. 2020; Ghanbari et al. 2015).

Fish in the family Poeciliidae are an interesting model for gut microbiome studies because they display a wide range of diets and many of the species are habitat generalists (Sullam et al. 2015; Meffe and Snelson 1989). The microbiomes of only three species of livebearing fish have been studied: the Trinidadian guppy ( *Poecilia reticulata* ), common mollies ( *Poecilia sphenops* ), and western mosquitofish ( *Gambusia affinis* ) (Sullam et al. 2012; Carlson et al. 2015), with only the Trinidadian guppy being examined in a natural setting. Here we describe the microbial communities in the digestive tract of a freshwater livebearing fish, *Heterandria formosa* , in several environmental settings using Illumina sequencing of the 16S rRNA gene. We quantified the associations between gut microbiome composition and diversity and variation in aquatic systems ranging from oligotrophic to near eutrophic. This is a critical first step in assessing whether the holobiont might be a unit of selection for local adaptation among these and other disparate microbial environments. It also serves as the foundation to determine to what extent the microbiota may affect adaptability of their hosts through internal bioremediation pathways as well as to uncover the potential for host‐adapted microbes to aid host adaptation to rapidly emerging environmental disturbances.

## 2 Methods

### 2.1 Study Species and Study Sites

**FIGURE 1:**
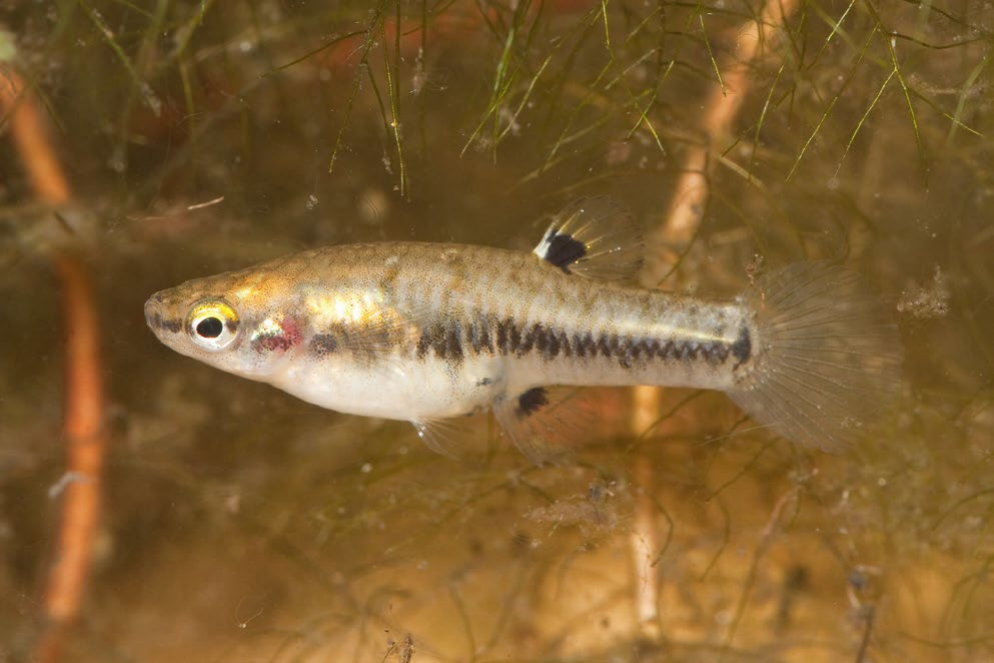
Female *Heterandria formosa* , photo courtesy of Pierson Hill.

*H. formosa* is a small freshwater livebearing fish in the family Poeciliidae found in the southeastern United States from southern North Carolina to eastern Texas (Figure 1). This species is the smallest known fish in North America with females ranging in size from 15 to 25 mm (Riehl and Baensch 1987). Individuals have particularly short generation times, as short as 2–3 months, making it well suited for experimental studies (Leips et al. 2000; Xie and Klerks 2004). Like all fish, they are in intimate contact with the abundant microbial communities in the aquatic environment (Mohammed and Arias 2015; Gomez et al. 2013).

Populations of *H. formosa* are found in the shallow littoral zone in a wide range of habitats in North Florida (Macrae and Travis 2014; Chaney et al. 2006). Populations of *H. formosa* are genetically distinct even at small spatial scales, consistent with little contemporary migration (Baer 1998; Soucy and Travis 2003; Schrader et al. 2011; Bagley et al. 2013). This means that each population is likely in contact with a unique set of microbial environments. Furthermore, unlike other well‐known poeciliids, like the eastern and western mosquitofish, *H. formosa* has undergone negligible human‐driven migration associated with mosquito control, suggesting that we are working with native populations (Jourdan et al. 2021).

**FIGURE 2:**
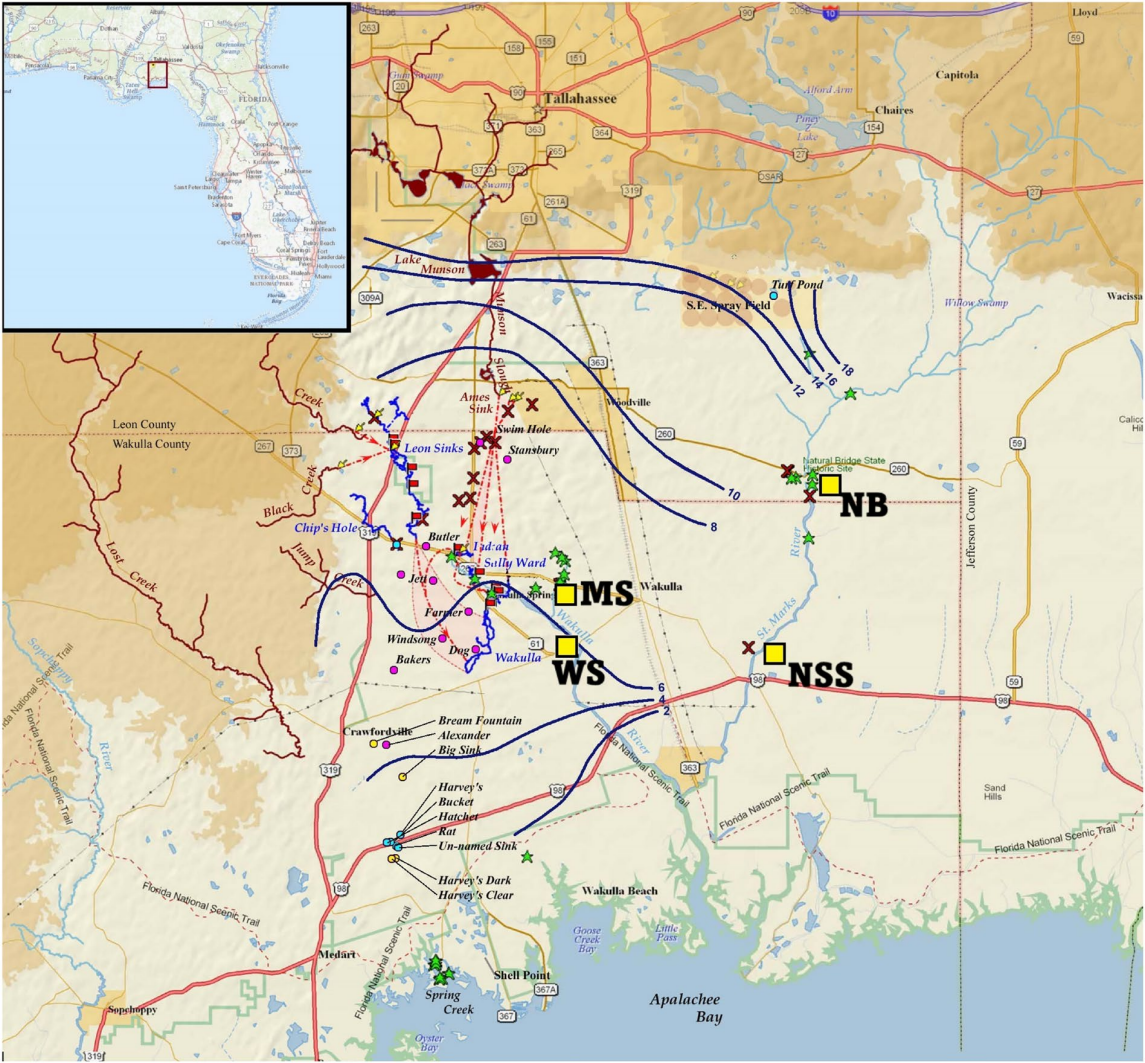
Map of sampling site locations for fish and water 16S rRNA microbial analysis relative to significant hydrologic features of the Florida aquifer in the Woodville Karst Plain, Florida; map courtesy of Florida Geological Survey.

**FIGURE 3:**
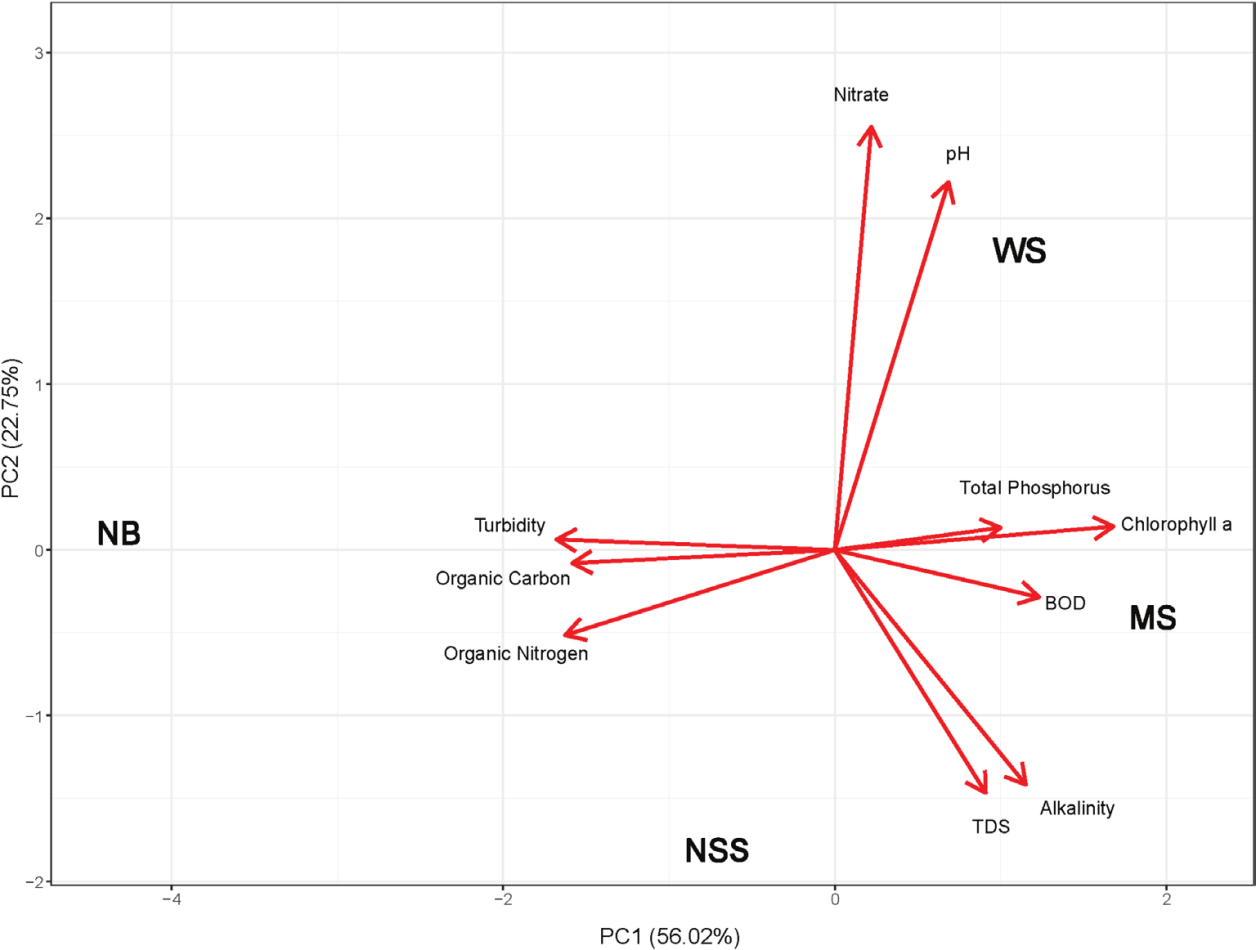
Principal component analysis (PCA) of our four Floridian spring sampling sites based on 10 water chemistry parameters from previously collected analyses (BOD, biochemical oxygen demand; TDS, total dissolved solids).

We focused our work on spring habitats, which are common in north Florida (Berndt et al. 1998; Scott et al. 2002). We selected four spring sites that varied in abiotic conditions for study (Leips and Travis 1999; Macrae and Travis 2014). The four spring sites selected for sampling are found south of Tallahassee, Florida, and each lies within 9 miles of each other (Figure 2). These sites included Natural Bridge Spring, NB (30.283352, −84.150986); Newport Sulfur Spring, NSS (30.206261, −84.178909); McBride Slough, MS (30.239326, −84.269539): and Wakulla Springs Upper Bridge, WS (30.213053, −84.26131). Spring sites range between first (NB and WS) and third magnitude (MS). Each site exhibits a unique combination of ecologically relevant water chemistry (i.e., carbon, nitrogen, and phosphorus) conditions (Figure 3; see also Dyer 2015; Kincaid et al. 2012).

There is little temporal variation in the abiotic features of north Florida spring systems (Leips and Travis 1999). It is well documented that Natural Bridge Historic Site (NB) is our most polluted site, with the highest instances of eutrophy and human pollution and is considered impaired according to the Florida Department of Environmental Protection (Barrios and DeFosset 2006; FDEP 2024). Our Wakulla Springs site (WS), in contrast, is our least polluted site, with water, after its emergence from the natural spring, running for two miles in the protected waters of Edward Ball Wakulla Springs State Park. Each site is found in the Woodville Karst Plain, a highly porous, productive limestone aquifer (Xu et al. 2019). Despite their separation, what we know about the underwater hydrology of the area and the highly productive limestone aquifers that this area sits upon suggests that a unified hydrological system is likely, but the degree to which these sites exchange water may be entirely dependent on precipitation patterns, among other environmental factors. Of the four sites, dye tracing projects done by the Florida Department of Environmental Protection and, specifically, the Geological Survey, suggest that Newport Sulfur Spring is likely most unique from the other sites because its sulphur‐rich connate water comes from deeper layers and mixes with the normal aquifer.

### 2.2 Data Collection

#### 2.2.1 Fish Collection/Sampling

In March of 2020, we sampled the four selected spring sites over 4 days. Fish were caught with scoop nets in shallow areas with submergent and emergent vegetation. We restricted our work to females, as adult sex ratios in *H. formosa* are female‐biased, often heavily so (Leips and Travis 1999; Leips et al. 2000) because of much higher mortality rates in males (see Felmy et al. 2021). This means that nutrient cycling and energy budgets largely run through females, giving us a better concept of host–microbiome interactions than we might with the use of males. The use of only females further allowed for greater sample uniformity, given published reports of differences between microbiomes of the sexes of other species. We kept females with a standard length of at least 20 mm for ease of dissection and quantity of digestive tract for eventual microbial DNA extraction. We collected 25 females per site for the eventual selection of 12 well‐dissected individuals.

#### 2.2.2 Fish Preservation and Dissection

After measuring each fish's standard length, we sacrificed fish via an ice‐cold bath (rapid chilling) on site according to IACUC protocols. Fish remained on ice for the return to the lab (> 45 min) to assure efficacy of euthanasia. After euthanasia, whole guts of individual *H. formosa* , made of a simple tubular digestive tract, were dissected from the oesophagus to the urogenital pore with sterile instruments based upon Sullam et al. (2015). Collected content included the entire digestive tracts as well as any associated gut contents. Digestive tracts that were torn in the process of dissection were not included in sequencing analysis.

#### 2.2.3 Gut Preservation and DNA Extraction

We stored the dissected digestive tracts in RNAlater at −20°C for no more than 2 weeks to allow thorough tissue penetration. RNAlater has been shown to successfully preserve both RNA and DNA for up to 1 month at room temperature and at 5°C. Within a week of cold storage, we isolated microbial DNA from each digestive tract separately with the QIAamp PowerFecal Pro DNA Kit (Qiagen, Hilden, Germany) in accordance with the manufacturer's protocol. Extracted DNA was stored at −80°C until sequencing.

#### 2.2.4 Fish Versus Site Water Microbial Community Comparisons

In addition to fish sampling, we collected water from each of the four sites in the locations from which we collected fish. Single samples of water were collected to prioritise site‐to‐site fish digestive tract comparison. We passed water through a 10 μm filtration on site, followed by 5 and 1 μm vacuum filtration in the laboratory to ensure removal of any contamination from our samples and to assure focus on the microbial communities present. We filtered 200‐mL volumes onto 0.2‐μm pore‐size cellulose ester filters (Whatman) to ensure capture of all associated communities, which we placed immediately into tubes for microbial DNA isolation. Microbial DNA was isolated from each filter separately with the QIAamp PowerFecal Pro DNA Kit (Qiagen, Hilden, Germany) in accordance with the manufacturer's protocol. Extracted DNA was stored at −80°C until sequencing. Extraction kits that optimise yields of pure microbial DNA from stool and gut samples were used as opposed to those that optimise yields from water to assure that we used comparable methods on water and digestive tract samples.

#### 2.2.5 Sequencing

The extracted DNA was submitted to the Florida State University Next Gen Sequencing Facility for amplicon library preparation and Illumina Miseq sequencing with the 600‐cycle v3 reagent kit of the V3–V4 hypervariable regions of the 16S rRNA gene using the following primers: 341F (5′‐TCGTCGGCAGCGTCAGATGTGTATAAGAGAC AGGA‐3′) and 785R (5′‐GTCTCGTGGGCTCGGAGATGTGTATAAGAGACAGCAGA‐3′). The V3 and V4 hypervariable regions were selected for their high reliability and for the ability for comparison to a plethora of other fish microbiome studies. The resulting sequencing data have been submitted to NCBI SRA under Bioproject PRJNA1228911.

#### 2.2.6 Bioinformatic Pipeline Information

Analysis and processing of our 16S rRNA gene reads were performed using the QIIME2 open access pipeline (v.2022.11). DADA2 was used for sequence quality control and filtering any phiX reads and chimeric sequences present in our samples. Paired ends were joined using QIIME. We trimmed the first 21 and 6 bases of the forward and reverse reads, respectively, and truncated sequences to 300 and 273 base pairs (forward and reverse) based upon the average quality scores for DADA2 analysis (Q‐threshold score of 26). Samples were then applied to amplicon sequence variants (ASVs). ASVs tagged as of non‐bacterial origin (Mitochondria, Chloroplasts, Archaea, Eukaryota, and Unassigned) were removed prior to any further analysis or manipulation.

### 2.3 Statistical Analyses

#### 2.3.1 Alpha Diversity

To quantitatively measure within‐sample community richness, we calculated Faith's Phylogenetic Diversity for each sample. This is a measure of community richness that incorporates phylogenetic relationships between amplicon sequence variants. We excluded from analyses 12 samples with less than 28,000 reads in order to gain accurate depictions of diversity while avoiding the need for rarefaction. To quantitatively measure within‐sample community evenness, we calculated Pielou's Evenness index for each sample. We analysed these measures of diversity with Kruskal–Wallis tests (*p* < 0.05) for all group and pairwise comparisons between sites.

#### 2.3.2 Beta Diversity

Beta diversity allows us to analyse community ASV similarity both within all the fish microbiomes in one site and between sites. We employed Principal Coordinates Analysis (PCoA) based upon Bray‐Curtis, Jaccard distances, and Unweighted UniFrac using QIIME2 to visualise both the quantitative and qualitative clustering of samples by site. We assessed the significance of these groupings with Permutational Analyses of Variance (PERMANOVAs). We additionally ran permutational tests of multivariate dispersion (PERMDISP) on the beta diversity indices at each site to test if the differences in spread of the samples were driving high beta‐diversity values between the sites.

#### 2.3.3 Taxonomic Analyses

We explored the taxonomic composition of each *H. formosa* digestive tract sample through the Greengenes database (v13.8) after creating 341F/785R based classifiers. We used analysis of composition of microbiomes (ANCOM) to identify which taxa contributed to the significant differences between microbial communities across sample groups. We conducted the ANCOM after further filtering of samples to remove low abundance digestive tract samples (p‐min frequency 17,785 paired‐end reads). Likewise, we removed observed amplicon sequence variants that appeared less than 10 times across all samples, along with observed sequence variants that only exist in a single sample for pairwise comparisons. We performed this analysis at the class‐genus levels in order to reveal any interesting interactions that might be hidden at higher classification levels.

#### 2.3.4 Mantel Tests and Canonical Correspondence

We looked for associations between five measures of water quality and the microbial communities of each population with Mantel analyses. We applied Mantel analyses on individual and scaled environmental distance matrices, based on Spearman's coefficient of correlation, within the Vegan (2.6‐2) package in R version (4.2.1) to test for significant associations. We ran 999 permutations for each analysis. We constructed microbial composition matrices using both Bray‐Curtis as well as Jaccard differences to test for both quantitative and qualitative associations. To further demonstrate the claimed associations between pH, chlorophyll a, nitrate, and microbial diversity, we performed canonical correspondence analysis (CCA) in R.

#### 2.3.5 Functional Profiling

Metagenomic function for each fish gut microbiome was predicted using PICRUSt2 (Phylogenetic Investigation of Communities by Reconstruction of Unobserved States) based on 16S rRNA gene sequences using a database of reference genomes. KEGG (Kyoto Encyclopedia of Genes and Genomes) Orthologs with relative abundances > 1% of all identified orthologs were included in our analysis. DESeq2 was used to conduct pairwise differential inferred gene representation analysis between fish at different sites based on the negative binomial distribution implemented. DESeq2 has the advantage of increased sensitivity on smaller datasets (< 20 samples per group) compared to the likes of edgeR. A log fold‐change > 1 and a *p*‐value < 0.001 were required to indicate significant differential abundance of KEGG Pathways between fish at different spring sites.

## 3 Results

### 3.1 Overall Community Composition

**FIGURE 4:**
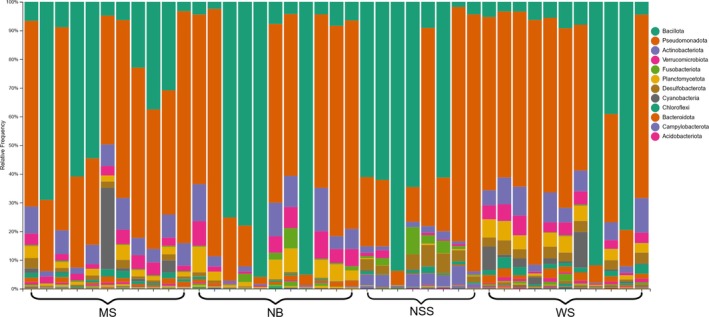
Taxonomic composition at the phylum level of digestive tract bacterial communities within *Heterandria formosa* . Results are based on 16S rRNA sequencing across 48 Least killifish. Accompanying legend shows the top 12 phyla, listed in order of prevalence throughout fish samples.

Approximately 6,000,000 paired‐end 16S rRNA gene V3–V4 region amplicon reads were obtained from the digestive tracts of *H. formosa* at the four spring sites, resulting in the identification of 36,249 amplicon sequence variants (ASVs). The largest average number of reads came from MS, 219,937, and the lowest average number of reads came from NSS, 57,152. Approximately 2500 of these ASVs were listed in the Unassigned, Archaea, or Eukaryota categories or were only classified to the domain level (Figure 4). Approximately 90,000 paired‐end 16S rRNA gene V3–V4 region amplicon reads were obtained from the water samples at the four spring sites, with the largest number of reads coming from NBW, 33,194, and the lowest number of reads coming from MSW, 12,374.

### 3.2 Comparison of Site Water Samples Versus Least Killifish Gut Bacteria

**FIGURE 5:**
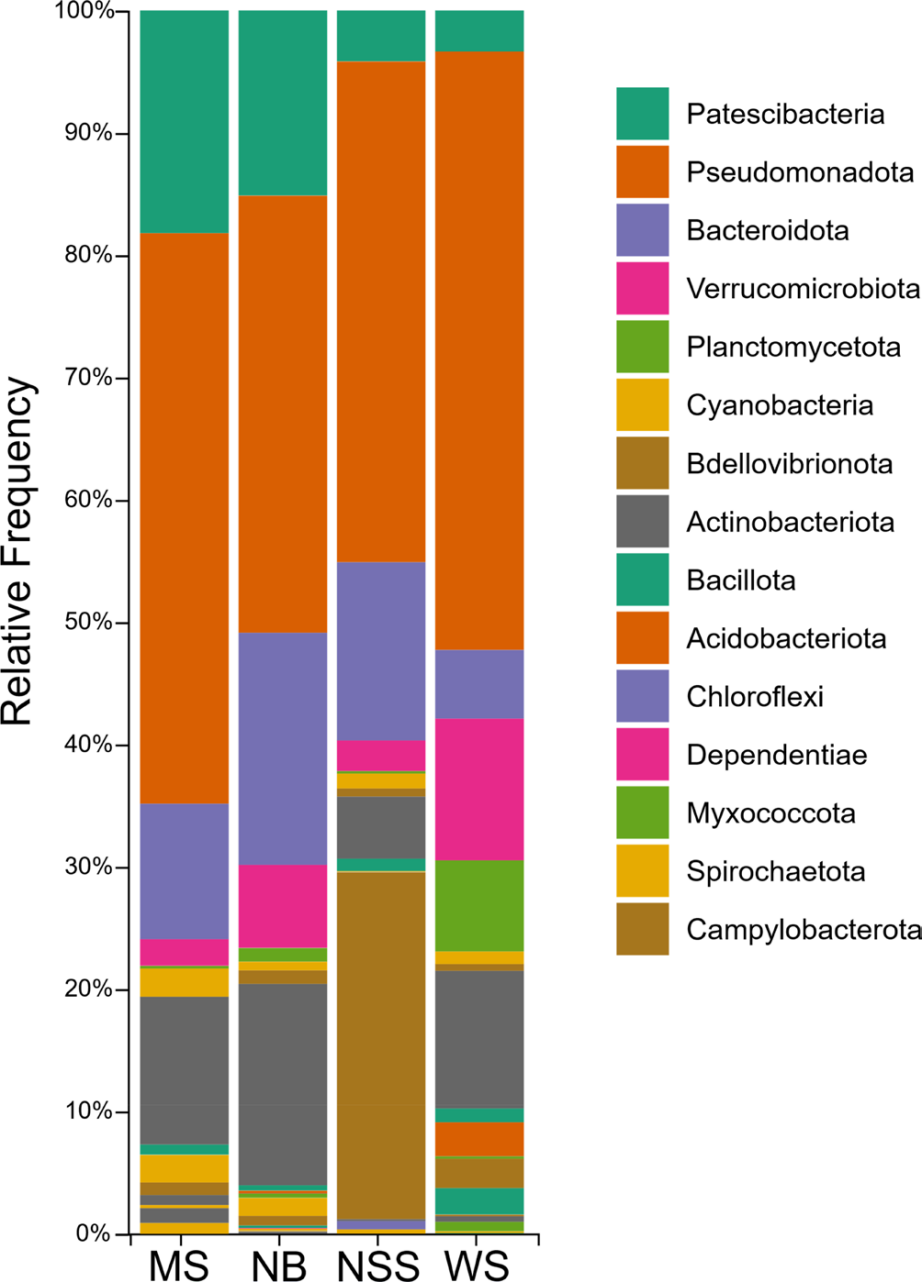
Taxonomic composition at the phylum level of water‐based bacterial communities at four sampled spring sites. Accompanying legend shows the top 15 phyla throughout the water samples.

The microbial composition of fish guts was very different from that of site water at all sites. There was a significant loss of classes within the phylum Patescibacteria from greater than 10% in water samples across all sites to less than 1% in fish digestive tract samples (Figure 5 compared with Figure 4; *p* < 0.0001, *t* = 4.174). The presence of Campylobacterota classes was also significantly reduced in fish samples (*p* = 0.0007, *t* = 3.66). We saw large decreases in overall Bacillota, formerly Firmicutes, classes including Bacilli (26.75%–0.54%) and Clostridia (4%–0.4%) in water samples compared to fish; however, the large standard deviations of these classes in fish drive an overall lack of statistical significance in those differences (*p* = 0.1260, fish SD = 33.27). The dominance of Bacillota in many fish samples is replaced by significant increases in Bacteroidia in our water samples, $\bar{x}$ = 11.78 (*p* < 0.0001, *t* = 4.174) and Actinobacteria, $\bar{x}$ = 9.81 (*p* < 0.0001, *t* = 4.174), which in fish represented 0.94% and 3.15% of samples respectively. Similarly, in the phylum Desulfobacterota, we see no significant changes between fish and water samples across sites, but significant changes when only considering NSS. Within NSS, we also see the presence of six Desulfobacterota classes in fish samples that are not present in any water samples.

### 3.3 Alpha Diversity Associated With Site Water Health

**FIGURE 6:**
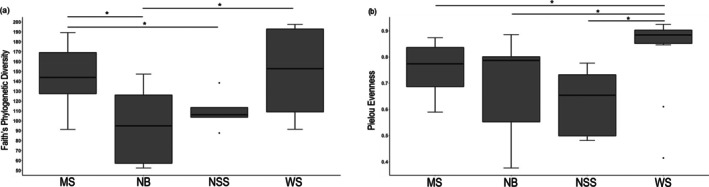
Comparison of alpha diversity indexes for digestive tract microbial communities between least killifish ( *Heterandria formosa* ) at four spring sampling sites. Diversity in the microbial communities was measured using Faith's Phylogenetic Diversity (a), Pielou's Evenness index (b). Asterisks above the boxplots indicate significant pairwise differences in alpha diversity between sampled sites (*p* < 0.05, Kruskal–Wallis test).

Overall, digestive tract microbial communities showed a relatively high degree of variability between individuals and sites. There were significant differences in richness among all *H. formosa* populations for all diversity metrics calculated (Faith's; *H* = 10.18, *p* = 0.01) (Figure 6a). Results from Shannon's diversity tests showed higher alpha diversity in terms of microbial richness at WS and MS compared to NB and NSS (Table 1a). In terms of microbial evenness, significant differences exist among all groups (Kruskal–Wallis) (*H* = 10.287, *p* = 0.016), with significant pairwise differences in alpha diversity detected by Kruskal–Wallis *H* test between WS and all other sites after Benjamini‐Hochberg correction for false discovery rates (Figure 6b, Table 1b). Additional diversity metrics suggest similar trends (Figures S1 and S2).

**TABLE 1:** Summary of Kruskal–Wallis (*H*) test for pairwise comparisons between four spring sites for digestive tract bacterial diversity analysis of *H. formosa* using Shannon's diversity (a) and Pielou's Evenness (b) metrics.

Kruskal–Wallis all groups		*H*	*p*‐value
Site 1	Site 2		
(A) Shannon diversity			
		10.62	0.014
MS (*n* = 12)	NB (*n* = 9)	3.96	0.047
MS (*n* = 12)	NSS (*n* = 5)	5.38	0.020
MS (*n* = 12)	WS (*n* = 10)	0.52	0.469
NB (*n* = 9)	NSS (*n* = 5)	0.75	0.386
NB (*n* = 9)	WS (*n* = 10)	5.61	0.018
NSS (*n* = 5)	WS (*n* = 10)	4.86	0.027
(B) Pielou's Evenness			
		10.29	0.016
MS	NB	0.73	0.394
MS	NSS	3.60	0.058
MS	WS	5.03	0.025
NB	NSS	0.75	0.386
NB	WS	6.00	0.014
NSS	WS	4.34	0.037

### 3.4 Compositional Differences Using Beta Diversity

**FIGURE 7:**
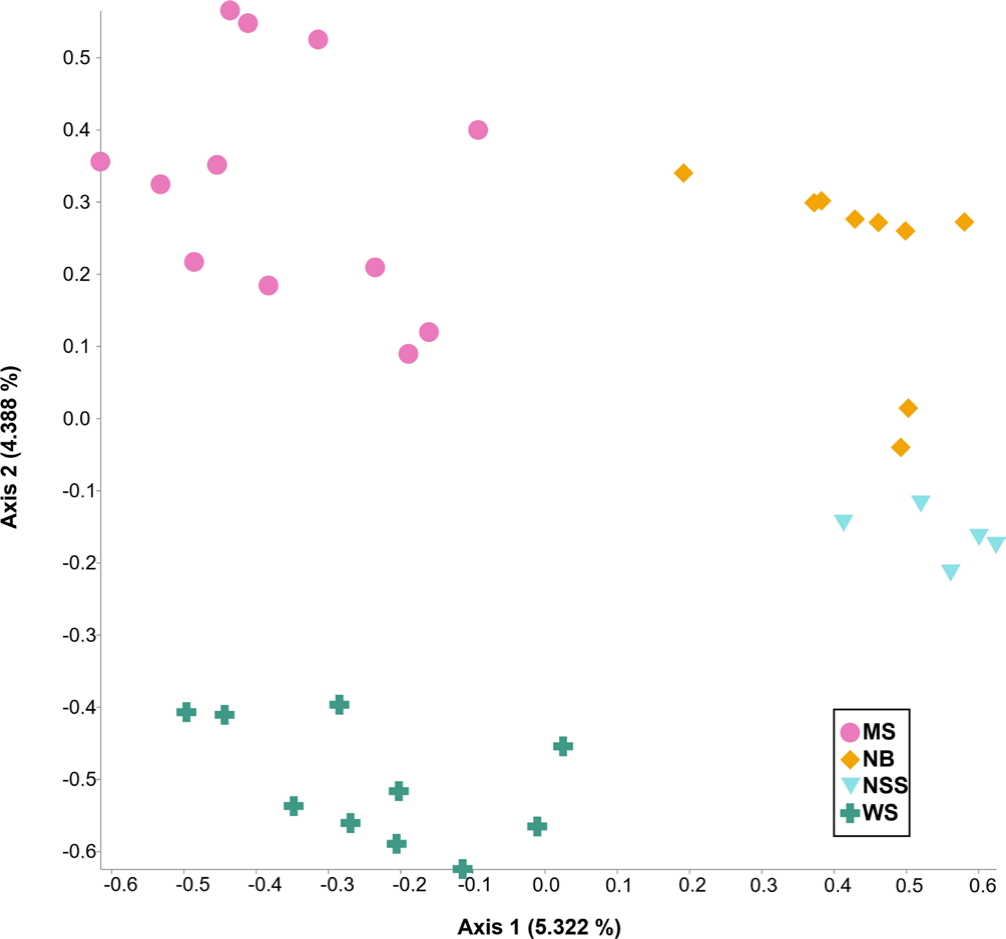
Similarity among the microbial communities found within the digestive tract of *Heterandria formosa* across sampling sites. Principal coordinate analysis (PCoA) based on Jaccard index analysis of microbial profiles.

**FIGURE 8:**
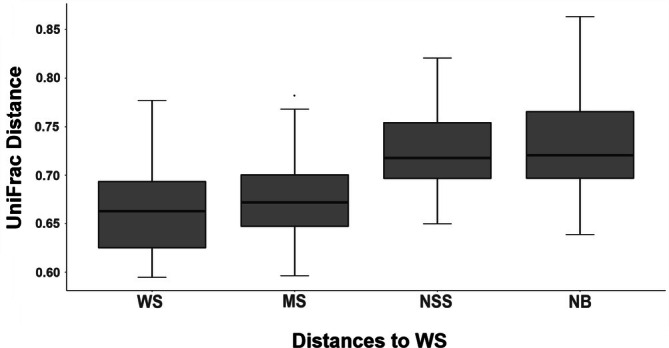
Comparison of beta diversity indices between digestive tract microbiomes of *Heterandria formosa* across sampling sites using unweighted UniFrac analysis of bacterial profiles.

There were significant differences between fish gut samples within a site and between samples across sites. PERMANOVA results suggest that fish gut samples for each of the four sites are drawn from microbiome distributions that are compositionally distinct among sites (pseudo‐*F* = 1.64, *p* = 0.001, Figure 7, Table 2). The lack of a significant PERMDISP test indicates that the differences in diversity between sites were not an artefact of different variances in each site (pseudo‐*F* = 4.77, *p* = 0.798). Despite significant results, PCoA plots based on unweighted UniFrac distances, which incorporate phylogenetic relationships between features, indicate more significant overlap in bacterial taxa among groups (pseudo‐*F* = 2.05, *p* = 0.001). Using UniFrac distances, we do note significantly greater phylogenetic similarity between more environmentally similar sites, which suggests that the microbiome of *H. formosa* could be influenced by the water chemistry of their environment. (Beta Difference Figures (Figure 8) and PCoA (Figure S3)).

**TABLE 2:** Summary of pairwise PERMANOVA results using Jaccard beta diversity distance comparisons between four spring sites for digestive tract bacterial diversity analysis of *H. formosa* .

Jaccard index			
PERMANOVA pairwise		Pseudo‐*F*	*p*‐value
Site 1	Site 2		
Group 1	Group 2		
MS	NB	1.70	0.001
MS	NSS	1.75	0.002
MS	WS	1.59	0.001
NB	NSS	1.44	0.002
NB	WS	1.66	0.001
NSS	WS	1.66	0.001

### 3.5 Microbiome Similarity Matches Water Chemistry

As samples became more dissimilar in terms of water chemistry, they also became more dissimilar in terms of microbial community composition. The cumulative environmental factors are weakly, but significantly, correlated with the microbial communities classified to the genus level (*r* = 0.1579, *p* = 0.0061). When tested individually, most water chemistry matrices have a relationship with both the Jaccard and Bray–Curtis dissimilarity matrix, but the relationships are weak (Tables 3 and S1). The pH of water and chlorophyll a concentration showed the strongest relationships with microbial taxa at all classification levels, with especially strong correlations at the genus level (*r* = 0.3159, *p* = 0.0001 and *r* = 0.2941, *p* = 0.0001). Overall reductions in Aeromonas, Cetobacterium, and the genus Gottschalkia within the class Clostridia alongside increases in the genus B1‐7BS within the Burkholderiales family as well as Mycoplasma were noted in tandem with increases in pH content. Total phosphorus and nitrate distances matrices showed lesser, yet significant parallels to overall microbial community structure. No significant correlation was seen with either beta‐diversity distance matrix for total nitrogen.

**TABLE 3:** Results of Mantel analysis using Jaccard beta diversity values testing the correlation between microbial communities and water chemistry parameters.

	Jaccard mantel tests											
	Scaled ALL		Total N		Total P		Nitrate		pH		Chlorophyll a	
	*r*	*p*‐value	*r*	*p*‐value	*r*	*p*‐value	*r*	*p*‐value	*r*	*p*‐value	*r*	*p*‐value
Class	0.005	0.168	0.019	0.305	0.153	0.005	0.082	0.070	0.191	0.0018	0.124	0.018
Order	0.089	0.053	0.014	0.346	0.184	0.001	0.124	0.018	0.252	0.0004	0.146	0.008
Family	0.107	0.028	0.022	0.293	0.195	0.001	0.142	0.010	0.272	0.0001	0.171	0.002
Genus	0.158	0.006	0.063	0.106	0.191	0.001	0.200	0.002	0.316	0.0001	0.225	0.001

Canonical correspondence analysis (CCA) revealed that microbial community composition in the gut of *H. formosa* was significantly structured by environmental variation among spring habitats. The first two constrained axes explained over 95% of the variation attributable to environmental factors, with CCA1 primarily reflecting a gradient from high chlorophyll a to high pH and nitrate concentrations (Figure S4). Samples from sites with similar water chemistry consistently clustered together, and phyla associated with eutrophic conditions, such as Pseudomonadota, Desulfobacterota, and Cyanobacteria, were enriched in these groups. Notably, Synergistota and Campylobacterota were dominant in the most eutrophic springs, suggesting potential roles in organic matter degradation and sulphur cycling. Conversely, taxa such as Bacillota and MBNT15 were associated with more oligotrophic or nitrate‐rich environments. Sites exhibiting higher eutrophic signatures harboured more diverse and compositionally distinct gut microbial communities, with CCA3 highlighting taxa potentially linked to enhanced metabolic capacity for nitrate and pollutant processing (i.e., Nitrospirota and Dadabacteria). These patterns support the hypothesis that environmental conditions, particularly those related to nutrient loading, shape host‐associated microbiota structure and function across *H. formosa* populations.

### 3.6 Taxonomic Analyses

Our results are similar in taxonomic makeup to the microbiomes of a wide variety of freshwater fish taxa (Tarnecki et al. 2017; Talwar et al. 2018; Kim et al. 2021) (Figure 4). The microbiota of *H. formosa* was dominated by Pseudomonadota, formerly Proteobacteria (47.11%) and Bacillota, formerly Firmicutes (31.09%) with smaller but significant amounts of Actinobacteria (5.49%). Additionally, before their removal in data cleanup prior to differential abundance testing with ANCOM, the phylum Cyanobacteria (18.50%) was dominant, likely associated with diatoms and soft algae in the periphyton that make up a large proportion of the *H. formosa* diet (Ye et al. 2014). We see significantly elevated representation (4.07%) of the anaerobic sulphate‐reducing bacteria, Desulfobacterota, in our Newport Sulfur Spring gut samples (*F* = 17.04, *p*‐value < 0.001) despite its low representation in NSS water samples (0.69%).

Across all sites, the digestive tract microbiota of *H. formosa* was consistently dominated by members of the classes Gammaproteobacteria (24.5%–37.0%) and Bacilli (21.2%–33.3%), belonging to the phyla Pseudomonadota and Bacillota, respectively. Alphaproteobacteria were also commonly detected, particularly at MS (17.2%) and NB (22.1%), while Actinobacteria (4.14%) were elevated in NB samples. Notably, Fusobacteriia were higher at MS (5.71%), and Campylobacteria were present at elevated levels in NSS fish samples (3.48%). WS and MS also featured Alphaproteobacteria in relatively high abundance (14.1% and 17.2%, respectively). Additionally, NSS samples were the only ones to exhibit appreciable levels of Clostridia (15.4%), while Planctomycetes (3.69%) were noticeably higher in WS samples.

At the order level, Mycoplasmatales (phylum Bacillota) was the most abundant across all digestive tract samples, particularly dominant at NB (32.04%) and NSS (25.80%). Members of Gammaproteobacteria were primarily represented by the orders Enterobacterales and Burkholderiales, both of which were found across all sites but showed particularly elevated representation in NSS (24.49% and 4.10%, respectively) and WS (8.26% and 17.33%, respectively). Rhizobiales and Rhodobacterales, orders within the Alphaproteobacteria, were also consistently detected, especially at MS and NB. The presence of Fusobacteriales was limited to MS (5.71%), while Clostridial orders, including Peptostreptococcales‐Tissierellales (8.70%) and Lachnospirales (5.27%), were unique to NSS samples.

### 3.7 ANCOM

Differential bacterial abundance analysis conducted using ANCOM comparing all fish between sites detected few highly significant compositional differences between populations at the class level. At NSS, we found elevated levels of not only bacterial taxa belonging to the phylum Desulfobacterota including Desulfobacteria, Desulfobulbia, and Desulfovibrionia, but also other potential sulphur‐reducing bacterial taxa, Campylobacteria (3.48%) compared to other sites (< 0.04%). This was, however, a significant reduction in the proportion of Campylobacteria found in NSS water samples (28.38%). Despite this significant reduction, Campylobacteria in NSS fish remained elevated in comparison to even all other site waters (MS: 1.03%, NB: 0.79%, WS: 2.38%).

Of the pairwise significant results at lower taxonomic levels, we could distinguish two broad associations of microbes and location. We found taxa that are indicators of high degradation status in river ecosystems; mainly in the orders Burkholderiales, Rhizobiales, and Rhodobacterales (Simon et al. 2017; Yang et al. 2019; Oren 2014), to be elevated in NB. The other group, which includes the bacterial families Rhodobacteraceae, Exiguobacteraceae, and Steroidobacteraceae, among others, are those that have been shown to be highly adaptable to variable environments including broad thermal, pH, salinity, and nutrient ranges (White et al. 2019; Mills et al. 2022). We found these variants at McBride Slough and Wakulla Springs.

### 3.8 Differential Gene Representation Across Sites

**FIGURE 9:**
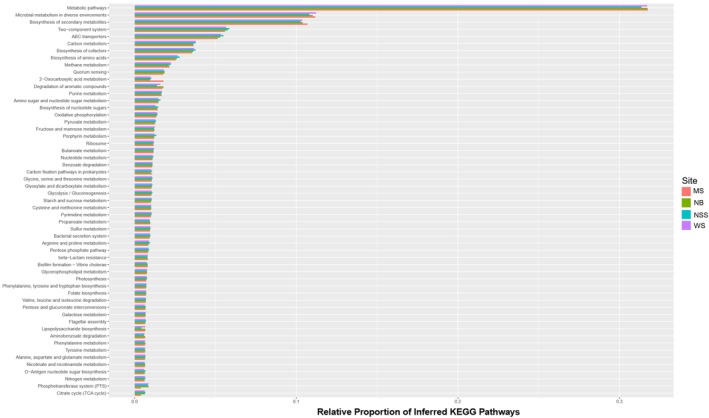
Relative proportion of the 55 most abundant PICRUSt2 inferred KEGG pathways associated with the digestive tract microbiota of *Heterandria formosa* .

**FIGURE 10:**
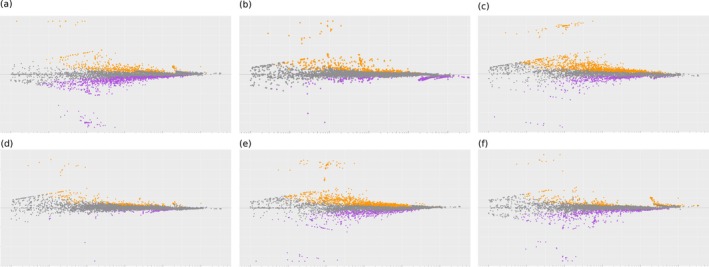
Scatter plots of log2 fold changes versus the mean of normalised counts for all pairwise comparisons between four sample sites [NSS‐WS (a), MS‐NB (b), MS‐NSS (c), MS‐WS (d), NB‐NSS (e), and NB‐WS (f)]. We used Phylogenetic Investigation of Communities by Reconstruction of Unobserved States (PICRUSt2) to predict metagenome function based on 16S rRNA sequences using a database of reference genomes and DESeq2 to determine metabolic capacity differences based on the negative binomial distribution. Orange dots indicate an upregulated pathway in the 2nd site in relation to the 1st. Purple dots indicate a downregulated pathway in the 2nd site in relation to the 1st.

The metagenomic functions inferred from PICRUSt2 were predominantly related to microbial metabolic pathways (> 30%) and biosynthesis of secondary metabolites (10%), followed by two‐component systems and ABC transporters (~5%) (Figure 9). Although not necessarily differentially expressed across sampled sites, significant portions of assumed metabolic functioning associated with carbon, nitrogen, purine and pyruvate metabolism exhibited relatively high representation in fish at all sites. Throughout our samples, we found pathways associated with metabolism of a variety of plant material (i.e., sucrose and starch), cell wall breakdown, and pathways that have the potential to provide carbon sources in anaerobic environments. We saw a much greater differential representation of fish gut microbial gene content between sites compared to the few differential species revealed in ANCOM analyses. Testing of the inferred KEGG pathways by DESeq2 revealed 514 out of 7889 KOs showing significant differences in microbial metabolic capacity (6.51% of total transcriptome) and even more than this that fell just outside of significance, given the conservative parameters that were set (Figure 10). All differentially represented KOs are summarised with Log2Fold and *p*‐values, including between 13 and 354 metagenomic pathways showing significant differential representation in pairwise comparisons of fish between the four sites (Log2fold‐change > 1 and a *p*‐value < 0.001) (Tables S2–S7).

The genes that showed significant pairwise greater representation in inferred functions within fish comprised groups of genes involved primarily in metabolic functions. These generally mimicked what was seen across all significant and insignificant KOs, which, in addition to microbial metabolism, were predominantly related to ABC transporters and biosynthesis of secondary metabolites. In pairwise comparisons, the gut microbiomes of NB fish only showed significantly elevated pathways in comparison to NSS.

Most significant differences consisted of Log2Fold changes with high values in functions associated with biofilm formation for *Escherichia coli* and *Pseudomonas aeruginosa* along with pathways for degradation of polycyclic aromatic hydrocarbons, styrene, dioxin, and other xenobiotics alongside more common metabolic pathways (Kowalchuk et al. 1994). One such group of functions was those associated with phenylalanine or phenylacetate degradation at our two most polluted spring sites, NB and MS. Other bacteria exhibited greater Log2Fold changes in assumed pathways associated with beneficial interactions with their aquatic hosts. Bacteria in MS fish had pathways associated with aiding fish in dealing with alkaline conditions by bringing H+ into the cell as well as cysteine and methionine metabolism. The NSS fish digestive tract microbiome was depleted of significant functions associated with sulphur metabolism and was enriched for assumed pathways associated with the *fumA* and *fumB* TCA cycles, which generate energy through the degradation of carbohydrates, fats, and proteins. The remaining pairwise comparisons revealed significant differences in various metabolic pathways including carbon, pyruvate, fatty acid, and sulphur metabolism, but no notable trends were seen across comparisons.

## 4 Discussion

### 4.1 Overview

We quantified the association between habitat trophic status and the digestive tract microbial communities of *H. formosa* in four spring habitats using Next‐generation sequencing of the 16S rRNA gene. We found considerable interindividual variability was detected, a finding that matches the in situ analysis of *P. reticulata* in Trinidadian streams, as well as much of the work in the fish digestive tract microbiome literature (Sullam et al. 2015). Despite this, we found differences between the microbial diversity of the digestive tract and that of the water in the habitat. We also detected subtle yet significant differences between the digestive tract microbial communities in overall diversity as well as community composition at a variety of taxonomic levels. From comparisons of diversity indices, we noted greater microbial phylogenetic similarity between more environmentally similar sites, supporting previous evidence that the microbiome of freshwater fish is associated with the water chemistry of their environment.

### 4.2 Overall Community Composition and Taxonomic Analyses

Much like prior research on Poeciliids (Sullam et al. 2012; Leonard et al. 2014), our samples were dominated by Pseudomonadota (Proteobacteria) (62.51%) and Bacillota (15.2%). In particular, our findings more closely mimicked fish in low predation environments in their dominance of Pseudomonadota. This result suggests the influence of host trophic level (Liu et al. 2016) or lack of predation stress (Zha et al. 2018) on microbiome composition. Our data on taxonomic composition are similar to those from studies of many freshwater and saltwater fishes but differ from the data reported for terrestrial vertebrates, which find a dominance of Bacillota and Bacteroidetes (Eichmiller et al. 2016; Zhang et al. 2016).

Most of the bacterial species within the Pseudomonadotal phylum are separated between Gammaproteobacteria and Alphaproteobacteria. The most dominant genus within the Gammaproteobacteria is Aeromonas, commonly known as a disease‐causing bacterium (Tomás 2012; Wang et al. 2016; Chen et al. 2019). While Aeromonas is present in all our samples, it appears most frequently in our NB samples (6/11 ≥ 5%), which was the most impaired site. Contrary to this trend, we see elevated levels of Cyanobacteria, which are typically associated with nutrient‐loaded, eutrophic waters, in the guts of fish in two of our healthier streams, WS and MS (Gallet et al. 2023). We know little about the role of cyanobacterial species in the fish microbiota and any effects they have on the health of the fish. The presence of these species at elevated levels in two sites suggests that further work on their functional roles in the fish guts would pay dividends.

Within the Bacillota phyla, the microbial communities across sites were dominated by Mycoplasmataceae phylotypes. Mycoplasmataceae has been found in a variety of freshwater and saltwater fishes (Ofek et al. 2021; Llewellyn et al. 2016). While species of this bacterium often are known for their pathogenic nature, high quantities in healthy fish suggest not only the existence of a fish‐associated Mycoplasma genus, but also the potential for symbiosis between host and bacteria (Bozzi et al. 2021; Sellyei et al. 2021).

In this same vein, one of the more striking taxa found in our digestive tract samples was those associated with sulphur reduction, including Desulfobacterota, which existed at elevated levels in NSS fish and in significantly higher amounts than exist in the NSS water. It is possible that the fish at NSS are using sulphur compounds in the water, which would be possible only through the symbiotic action of their bacterial associates. In addition to elevated levels of these bacteria at NSS, we also saw small amounts at the two downstream sites WS and MS, which further suggests the interconnected microbial pathways present in the Woodville Karst Plain.

### 4.3 Comparison of Site Water Samples and Least Killifish Gut Bacteria

We acknowledge the limitation of the use of single samples of water used for comparison, a decision that was made to prioritise site‐to‐site fish digestive tract comparison over water to digestive tract comparison. Further, more robust, potentially controlled comparisons of water chemistry on digestive tract microbial communities would further elucidate the impact host environment has on these organisms.

The ASVs present in the digestive tracts were also present in our water samples but at significantly lower concentrations. This might suggest that the site water microbiomes play a role in seeding least killifish digestive tracts (Nayak 2010). The minimal overlap between our water microbial samples and the digestive tract samples suggests that our fish appear to have the capacity to create an internal environment from a select consortia of microbial communities different from the external environment and even from their food. This is especially true in the significant loss of Patescibacterial classes. Patescibacteria are prevalent in groundwater environments and have limited genetic material. While these bacteria are able to adapt to changes and thrive in their aquatic environment, a majority of their genes are associated with growth and reproduction. While this simplicity works for this phylum on its own, it appears not to be beneficial for host–microbial symbiosis and therefore our fish may be actively selecting against it in their digestive tracts.

### 4.4 ANCOM

The low number of significant pairwise differences in compositional diversity suggests the presence of a core microbiome in the digestive tract of *H. formosa* at each site that is fulfilling a fundamental role regardless of what water chemistry environment they and their host are experiencing. It is possible that small deviations of the microbiome at each site and between individuals may be meaningful, functionally, but are not large enough to appear in pairwise comparisons. Within those taxa that do show significant pairwise differences, our results suggest that water quality may be driving the microbial composition of the digestive tract of *H. formosa* . Bacterial taxa that are found to be dominant in the digestive tracts of fish at our most polluted site, Natural Bridge, show frequent association with degraded habitats, often associated with parasitic or opportunistic potential (Simon et al. 2017; Dahal et al. 2021; Yang et al. 2019). Alternatively, the presence of highly adaptable bacteria at healthier sites suggests the potential for coevolution between bacteria and host under non‐stressful conditions that allow for these interactions (Mills et al. 2022).

### 4.5 Alpha and Beta Diversity

Overall, digestive tract microbial communities showed a high degree of variability between individuals and sites, which is often noted in a wide range of fish microbiota studies (Sullam et al. 2012; Tarnecki et al. 2017; Talwar et al. 2018). Because of this, we implemented several different measurements of alpha and beta diversity. The findings of alpha and beta diversity testing supported the two main results of our log‐ratio ANCOM testing. Between fish in different populations, there were significant differences in richness among all *H. formosa* populations. Results showed higher alpha diversity in terms of microbial richness at WS and MS compared to NB and NSS. This result reinforces our predictions that WS would prove one of our healthier sites and NB as one of the least healthy. Bacterial diversity of NSS is low, but as NSS is a unique higher sulphur environment, low diversity may be due to the dominance of more specialised bacteria that are able to thrive in these conditions.

In terms of microbial evenness, significant differences exist among all groups with significant pairwise differences in alpha‐diversity detected between WS and all other sites. A strong positive correlation has been found between bacterial species evenness and overall health or performance due to increased system functionality (Haig et al. 2015; Zhang et al. 2021; Choudhary and Mahadevan 2022). This further suggests that the health of an ecosystem is not only beneficial to the host directly, but also beneficial, indirectly, through the microbiota that these hosts are therefore able to acquire and retain.

How much difference we saw between sites in the digestive tract biota depended upon whether we took phylogenetic relationships into account. With analyses of the simple beta‐diversity measures that do not incorporate phylogenetic relationships, we found a number of significant differences between the digestive biota in different sites. This could have been driven by noted differentiation in water chemistry as well as subtle differences in diet. When considered in the context of weighted phylogenetics, we saw fewer pairwise significant comparisons between sites. This suggests that while fish at these sites host a taxonomically similar core microbiome at higher classification levels, at lower levels we observed subtle, yet significant changes based upon individual site characteristics.

### 4.6 Microbiome Similarity Matches Water Chemistry

Many studies have demonstrated that environmental factors are important in influencing bacterial community composition, particularly those that relate to habitat and resource quality (Sullam et al. 2012; Ghanbari et al. 2015). We found that the greater the environmental distance between populations, the more likely those populations are to have disparate gut microbial communities. The *r*‐values associated with our Mantel correlations indicated larger effect sizes when each variable was tested individually compared to their being examined together, which suggests that certain water chemistry parameters influence our microbiota more than others. These data alone do not allow us to discern why some variables were more important than others nor whether that importance stemmed from characteristics of the aquatic environment or features of local diets. Local differences in water chemistry could influence the microbial communities present in the water around the host, and therefore the pool of bacterial species likely to colonise the digestive tract. Alternatively, local features of water could influence species composition of the periphyton on which these fish graze, which could be determining the composition of their digestive biota. Conclusions of water chemistry influence and the predominant genera with respect to water chemistry may be drawn from our principal component analysis (PCA) of our four Floridian spring sampling sites (Figure 3) in combination with overall taxonomic community composition results (Figure 4).

The weak but significant correlation between water pH at a site and the beta‐diversity composition is an especially interesting association. This result is similar to those from other studies that showed changes in microbiota based upon pH changes. This species occurs in habitats that vary widely in pH level (Leips and Travis 1999; Macrae and Travis 2014). Individual *H. formosa* grow faster at lower pH, but there is no evidence for local adaptation to pH level (Hale and Travis 2015). Taken together, these results suggest that the wide pH tolerance of this species might be influenced by the microbiota within their digestive tracts (Sylvain et al. 2016; Fierer et al. 2012).

Our CCA findings demonstrate that environmental variation across spring habitats plays a significant role in shaping the gut microbiota of *H. formosa* , supporting the concept of environmentally mediated host–microbiome interactions. The distinct clustering of microbial communities by site, along with strong associations between microbial taxa and environmental variables such as chlorophyll a, nitrate, and pH, suggests that water chemistry exerts selective pressure on gut community structure. Enrichment of taxa such as Desulfobacterota and Campylobacterota at eutrophic sites points to potential microbial contributions to the degradation of organic pollutants and sulphur compounds, which may offer functional benefits to the host under nutrient‐stressed conditions. These results highlight the adaptive potential of host‐associated microbial communities and underscore the importance of conserving environmental conditions that support beneficial microbial symbioses in freshwater fishes facing rapid ecological change.

### 4.7 Differential Gene Representation Across Sites

Much of our functional profiling categorisation is speculative due to the vague categorisation of functional profiling within aquatic environments and within aquatic hosts. Additionally, while PICRUSt2 provides general predictions on metabolic pathways, we acknowledge that more direct methodologies such as shotgun metagenomics should be used in the future to verify the metabolic pathway predictions from the programme. In our Natural Bridge digestive tract samples, we see assigned functional pathways associated with polycyclic aromatic hydrocarbons, styrene, and dioxin degradation that appear at no other sites we sampled. This is the only site where we see such a high abundance of assumed pathways associated with human pollution. This includes the assumed pathways associated with phenylalanine or phenylacetate degradation at our two most polluted spring sites, which gives us insight into the natural remediation of man‐made environmental contaminants, but further work is needed to understand these and other roles in conjunction with their host. Furthermore, this pathway occurs in various pathogens, where its reactive early intermediates may contribute to virulence; therefore, determining if it has a beneficial role within the host is unknown (Teufel et al. 2010).

The lack of response of bacterial inferred pathways to eutrophic conditions was unexpected. This is particularly true for nitrogen; sites with elevated nitrogen sources were not significantly elevated in the number of inferred pathways involved in cellulose, xylan, starch, and sucrose degradation, an association shown in previous work (Amend et al. 2016). Instead, pathways associated with the metabolism of a variety of plant material (i.e., sucrose and starch), cell wall breakdown, and pathways that have the potential to provide carbon sources in anaerobic environments were seen throughout our differential gene representation analysis (Condemine and Robert‐Baudouy 1991; Fouet et al. 1986). In addition, our PICRUSt2 results suggest that NSS fish are depleted of functions associated with sulphur metabolism, a result that contrasts with what we detect in our taxonomic analysis. Further analysis of taxa within the Desulfobacterota phylum shows most major elevated classes, including Desulfobulbia and Desulfomonilia, to be associated with sulphur reduction (Waite et al. 2020; Murphy et al. 2021). This result may be driven by the elevation of another sulphur‐reducing class, Desulfobaccia, at MS and WS, but not at NSS, or the significant elevation of sulphur‐reducing bacteria in just over half of our NSS samples. Alternatively, our more stringent parameters when running PICRUSt2 over ANCOM might drive the disparity between results.

The one clear association of differences in assumed pathways with water chemistry involved the presence of pathways associated with pH regulation. This supports our results from the Mantel analyses that pH variation was the largest driver of microbial compositional change. Fish and bacteria in MS appear to be experiencing more alkaline conditions than our water chemistry tests indicate. Elevated pathways have been documented in importing H+ into cells and helping them deal with alkaline conditions (Guffanti et al. 2002; Putnoky et al. 1998). pH regulation also has the potential to create environments that suppress dangerous bacteria, which would further benefit bacteria and host (Dawood and Koshio 2019).

We also speculate that elevated pathways associated with cysteine and methionine metabolism are being carried out by associated bacteria. Methionine is an essential amino acid that is often added as a supplement in fish feed (Wang et al. 2023) which in high doses promotes protein utilisation and immune system function (Alam et al. 2000; Tang et al. 2009), likely due to increased bacterial activity (Ye et al. 2014), and whose deficiency is linked to poor growth and survival (NRC 2011; Shoaib et al. 2022). Alternatively, in other fish and animals, methionine restriction is known to produce lifespan extension and has been more recently investigated for its role in improving metabolic health, leaving further ambiguity into the exact role that these metabolic pathways may play (Latimer et al. 2018). Much like pathways associated with methionine and cysteine metabolism, we noted an abundance of assumed pathways whose role, beneficial or detrimental, is unclear. A knowledge gap exists in understanding the specific functioning of the digestive tract microbiome, partly due to the complexity and variability in the ecology of freshwater fish digestive tracts and owing to the abundance of bacterial taxa that have yet to be categorised (Perry et al. 2020; Egerton et al. 2018). Future studies aiming to use proteomic or metabolomic approaches to understanding the role microbial communities play in the digestive tracts of aquatic organisms are called for and would further the goal of a comprehensive approach to ecosystem conservation.

## 5 Conclusion

The study contributes to the growing literature on microbial associations by expanding our knowledge of the digestive tract microbiome of fish to a non‐aquaculture associated, non‐invasive species. We found, first, that changes in the overall health of spring sites influenced both overall diversity and composition of digestive tract microbial communities. Second, despite the dominance of taxa seen in other freshwater fish studies, our findings bring to light some of these particular bacterial taxa and pathways that have the potential to play critical roles in the bioremediation of specific nutrient‐stressed conditions and call for future research on the exact contribution of these features to the improved function of the aquatic holobiont. Alternatively, we note species in both water and digestive tract samples that have the potential to disrupt symbiotic relationships between host and bacteria by acting as pathogens and causing disease. We also find taxa that have the potential to facilitate the host's performance in eutrophic conditions. Our research provides insights in both the aquaculture and natural contexts, understanding how pollution and eutrophication affect microbiota and suggesting the importance of comprehensive conservation solutions to prevent their negative impacts (Li et al. 2017; Jung‐Schroers et al. 2016). Similarly, by better understanding the components that help shape these variable digestive microbial communities, we can gain insight into the potential for host adaptation and its impacts on sustaining local biodiversity.

## Author Contributions

**Benjamin D. Pluer:** conceptualization, investigation, writing – original draft, funding acquisition, methodology, validation, visualization, writing – review and editing, software, formal analysis, project administration, data curation, resources, supervision. **Joseph Travis:** funding acquisition, writing – review and editing, supervision, resources.

## Conflicts of Interest

The authors declare no conflicts of interest.

## Supporting information


**TABLE S1:** Results of Mantel analysis using Bray Curtis beta diversity values testing the correlation between microbial communities and water chemistry parameters.

**TABLE S2:** Pairwise KO [MS‐NB].
**TABLE S3:** Pairwise KO [MS‐NSS].
**TABLE S4:** Pairwise KO [MS‐WS].
**TABLE S5:** Pairwise KO [NB‐WS].
**TABLE S6:** Pairwise KO [NSS‐NB].
**TABLE S7:** Pairwise KO [NSS‐WS].

## Data Availability

The data that support the findings of this study are openly available on NCBI SRA under Bioproject PRJNA1228911.
