# Supplementary material for: The Digestive Microbiome Diversity of the Least Killifish, Heterandria formosa , and Its Implications for Host Adaptability to Varying Trophic Levels
Source: Environ Microbiol Rep. 2025 Aug 12;17(4):e70164. doi: 10.1111/1758-2229.70164 (PMC12342959; doi:10.1111/1758-2229.70164)
Supplement: Supplementary file 1 — TABLE S1: Results of Mantel analysis using Bray Curtis beta diversity values testing the correlation between microbial communities and water chemistry parameters. [file EMI4-17-e70164-s002.docx]

Supplemental Information for:

The Digestive Microbiome Diversity of the Least Killifish, *Heterandria Formosa,* And Its Implications in the Nutrient Loading Stress Abatement in Freshwater Fish

Benjamin Pluer^1^ and Joseph Travis^1^

^1^Department of Biological Science, Florida State University, Tallahassee, FL, USA

bdpluer@bio.fsu.edu, ORCID: 0000-0002-0835-5119

travis@bio.fsu.edu, ORCID: 0000-0002-2419-5020

|  | **BRAY CURTIS MANTEL TESTS** | | | | | | | | | | | |
| --- | --- | --- | --- | --- | --- | --- | --- | --- | --- | --- | --- | --- |
|  | **Scaled ALL** | | **Total N** | | **Total P** | | **Nitrate** | | **pH** | | **Chlorophyll a** | |
|  | **r** | **p-value** | **r** | **p-value** | **r** | **p-value** | **r** | **p-value** | **r** | **p-value** | **r** | **p-value** |
| **Class** | 0.02251 | 0.28116 | -0.031 | 0.7749 | 0.04742 | 0.1476 | 0.06438 | 0.1001 | 0.01522 | 0.0062 | 0.1068 | 0.0239 |
| **Order** | 0.08643 | 0.0485 | -0.0036 | 0.4749 | 0.09701 | 0.0322 | 0.1291 | 0.0162 | 0.2364 | 0.0007 | 0.1332 | 0.0097 |
| **Family** | 0.1009 | 0.0277 | 0.00914 | 0.3763 | 0.1182 | 0.0142 | 0.1384 | 0.0069 | 0.2419 | 0.0003 | 0.1546 | 0.0042 |
| **Genus** | 0.1588 | 0.0049 | 0.05734 | 0.1109 | 0.1207 | 0.0146 | 0.2028 | 0.0019 | 0.2941 | 0.0001 | 0.2025 | 0.0011 |

*Table S1: Results of Mantel analysis using Bray Curtis beta diversity values testing the correlation between microbial communities and water chemistry parameters.*

*Table S2: Pairwise KO [MS-NB] See attached Excel document.*

*Table S3: Pairwise KO [MS-NSS] See attached Excel document.*

*Table S4: Pairwise KO [MS-WS] See attached Excel document.*

*Table S5: Pairwise KO [NB-WS] See attached Excel document.*

*Table S6: Pairwise KO [NSS-NB] See attached Excel document.*

*Table S7: Pairwise KO [NSS-WS] See attached Excel document.*

**Supplementary Figures**

*
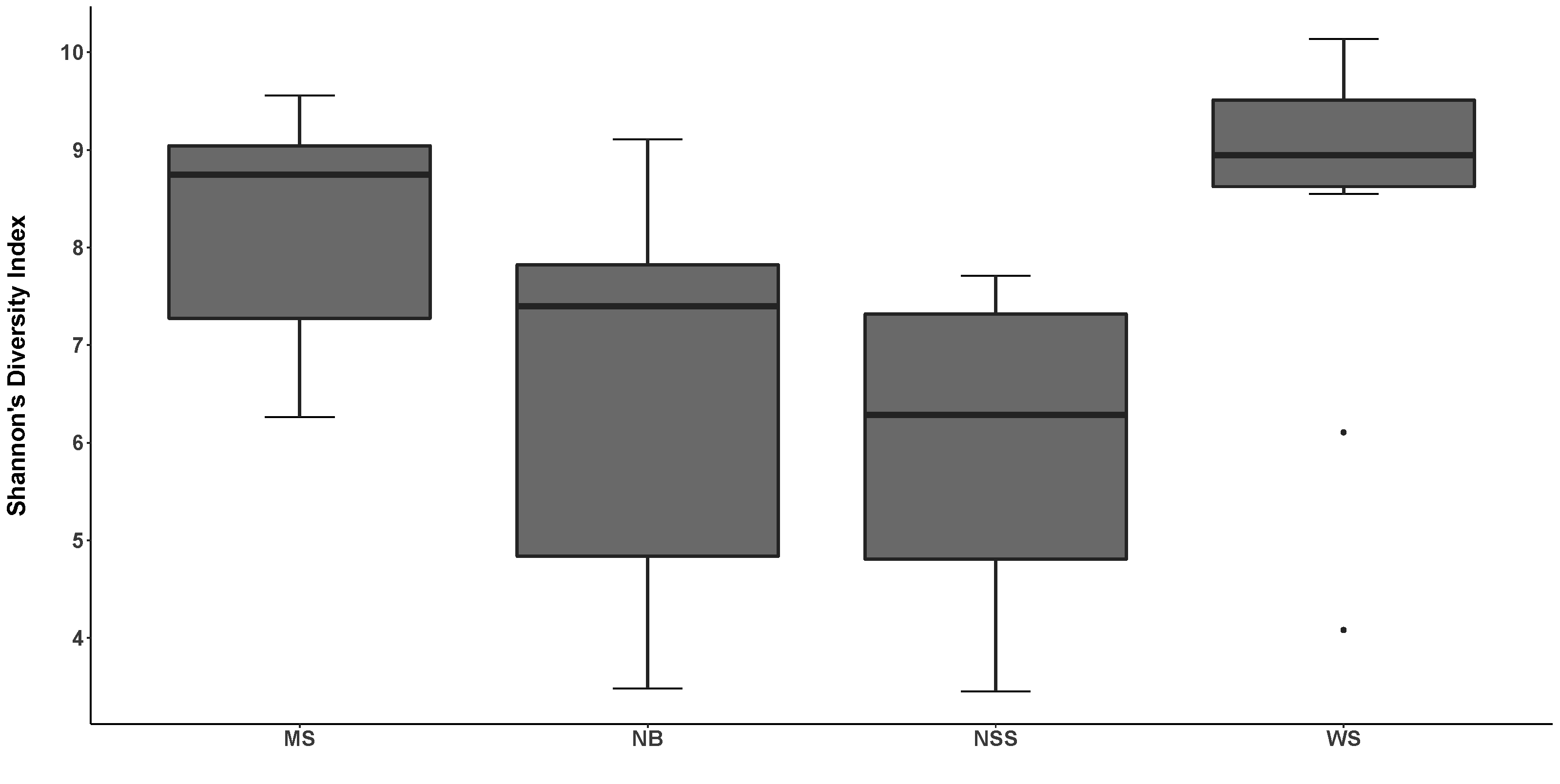
*

*Figure S1:* Comparison of Shannon Alpha Diversity for digestive system microbial communities between least killifish (*Heterandria formosa*) at four spring sampling sites. Asterisks above the boxplots indicate significant pairwise differences in alpha diversity between sampled sites (p < 0.05, Kruskal-Wallis test).

*
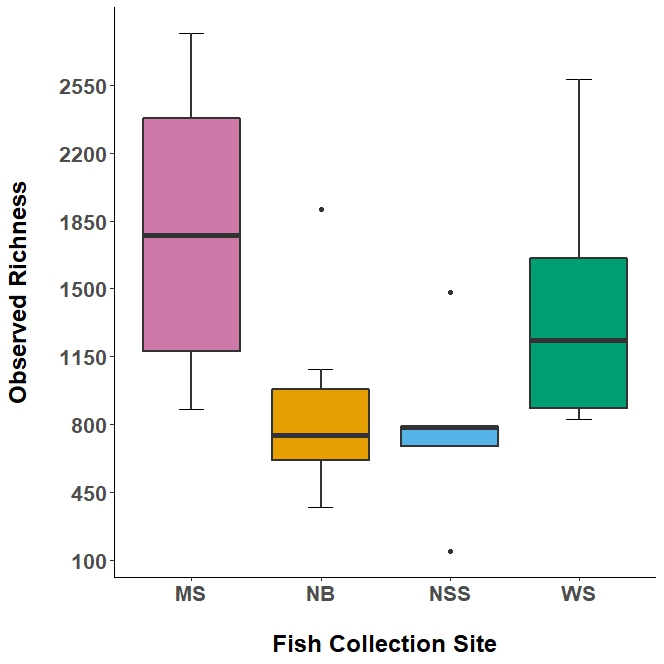
*

*Figure S2:* Comparison of Observed Features Alpha Diversity for digestive system microbial communities between least killifish (*Heterandria formosa*) at four spring sampling sites. Asterisks above the boxplots indicate significant pairwise differences in alpha diversity between sampled sites (p < 0.05, Kruskal-Wallis test).

*
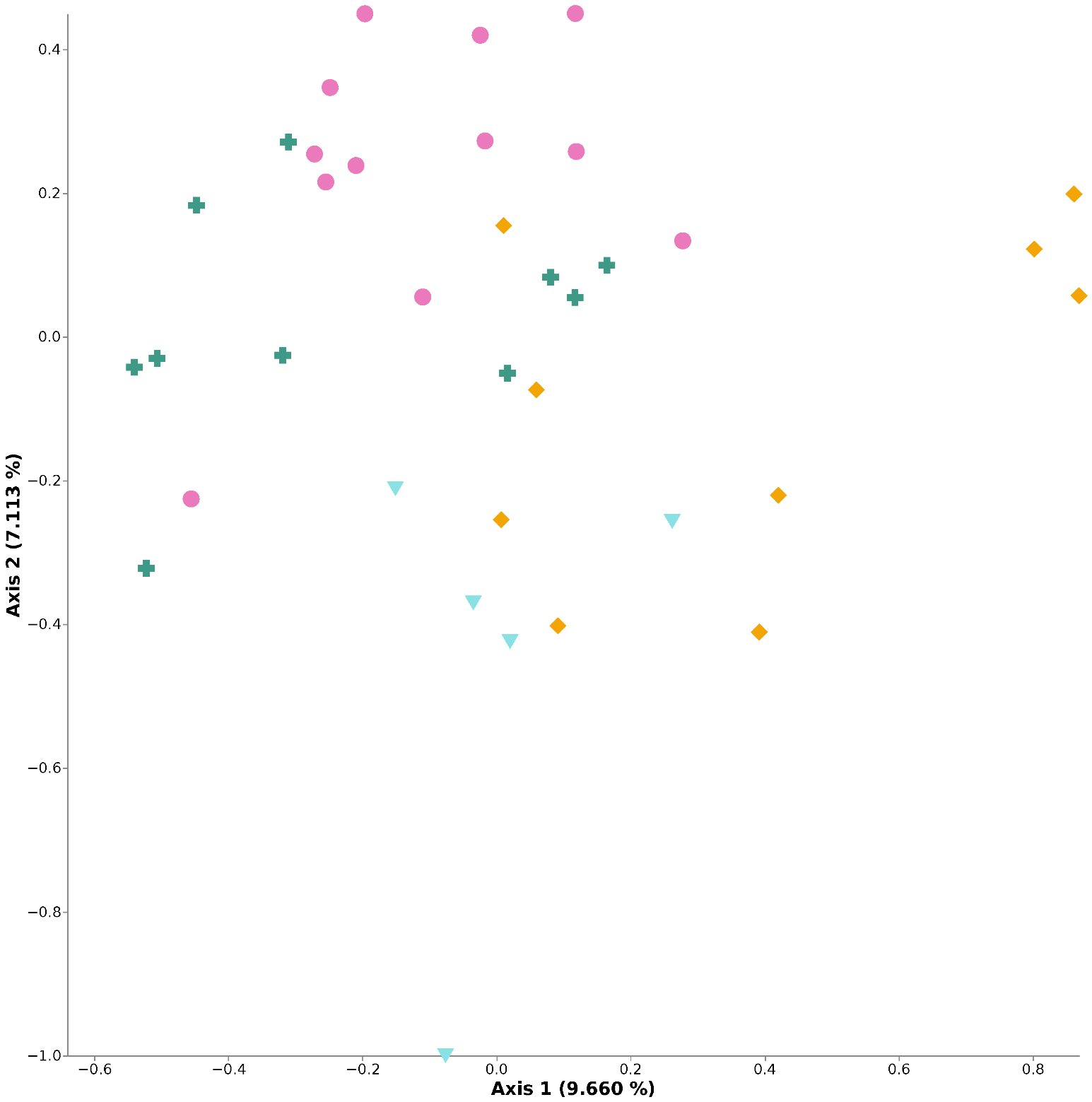
*

*Figure S3:* Similarity among the microbial communities found within the digestive system of *Heterandria formosa* across sampling sites. Principal coordinate analysis (PCoA) based on *Unweighted UniFrac PCoA* analysis of microbial profiles.


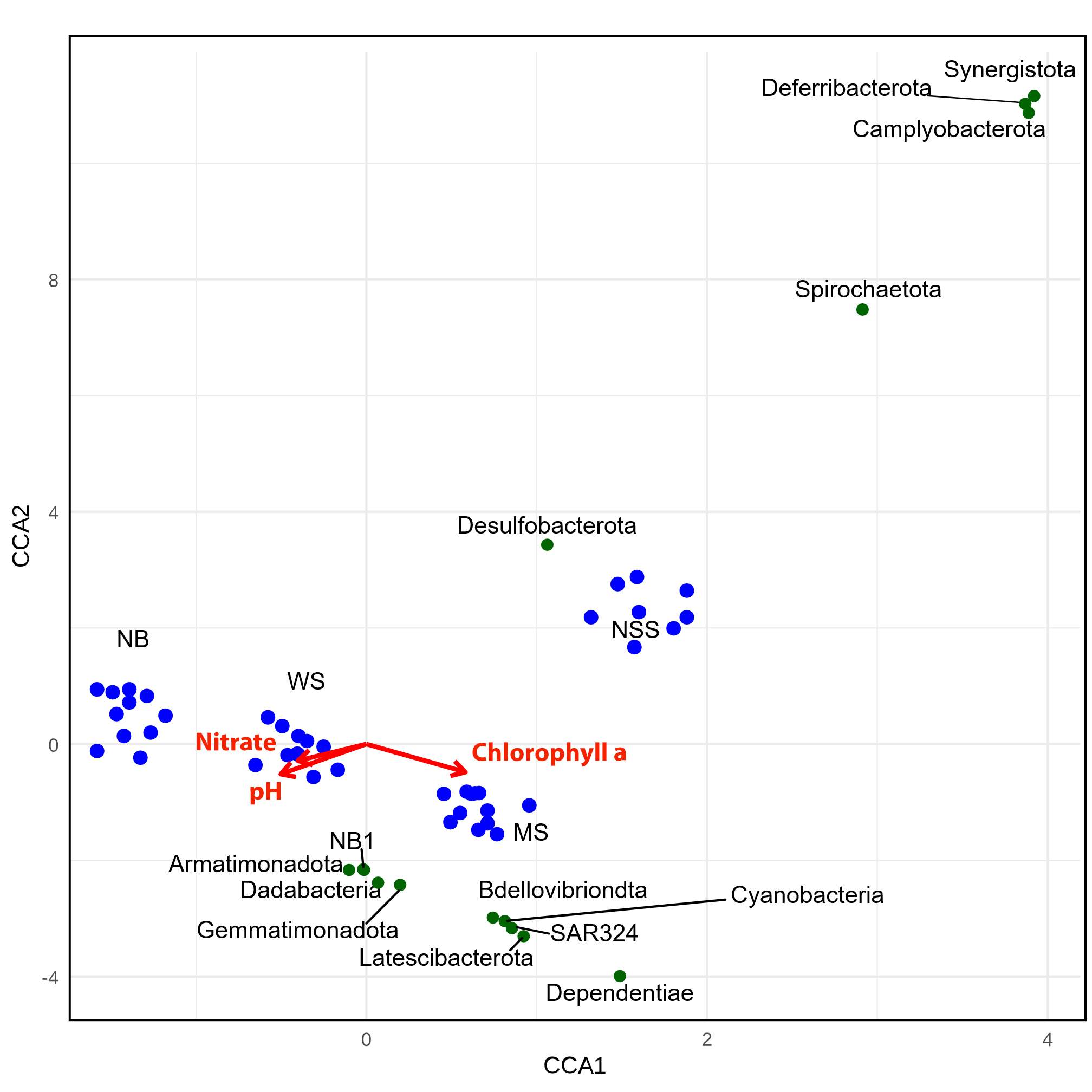


*Figure S4:* Canonical Correspondence Analysis (CCA) biplot revealing the relationships between strongly correlated environmental variables and fish microbiomes and specific microbial phyla. Dots represent individual fish microbiome scores (blue) and microbiome phyla scores (green). Red arrows indicate the strongly correlated environmental variables, with arrow direction and length representing the gradient and strength of their influence on species composition. The proximity of species or sites to arrows suggests associations with specific environmental gradients, highlighting how environmental factors structure community composition.
